# The Effectiveness of Budesonide Nasal Irrigation in Patients with Allergic Rhinitis

**DOI:** 10.21315/mjms2022.29.1.4

**Published:** 2022-02-23

**Authors:** Muhammad Nu’aim Ishak, Norasnieda Md Shukri, Ramiza Ramza Ramli

**Affiliations:** 1 Department of Otorhinolaryngology-Head and Neck Surgery, School of Medical Sciences, Universiti Sains Malaysia, Kelantan, Malaysia; 2 Faculty of Medicine, Universiti Sultan Zainal Abidin, Medical Campus, Terengganu, Malaysia

**Keywords:** allergic rhinitis, nasal irrigation, budesonide, sino-nasal outcome test, endoscopy

## Abstract

**Background:**

Allergic rhinitis (AR) is a long-standing disease and has been shown to cause significant impairment in patients’ quality of life. Saline nasal irrigation is a proven adjunct in the treatment of AR. The addition of steroid to the saline solution can provide local steroid effect and increase the effectiveness of this technique. Our study aimed to determine the effectiveness of budesonide nasal irrigation as an adjunct to the treatment of AR, compared with saline nasal irrigation.

**Methods:**

This was a randomised controlled study involving 99 patients diagnosed with AR, half of whom were treated with saline nasal irrigation and the other half with budesonide nasal irrigation. Parameters measured include the Sino-Nasal Outcome Test (SNOT-22) questionnaire, endoscopic nasal examination findings and blood eosinophil count.

**Results:**

Patients treated with budesonide nasal irrigation had significant improvement in total SNOT-22 score (*P* < 0.001) and improvement in the endoscopic nasal examination findings, such as nasal mucosa oedema and secretions (*P* < 0.001). However, there was no significant improvement of blood eosinophil count in patients treated with either budesonide or saline nasal irrigation.

**Conclusion:**

Budesonide nasal irrigation is effective as an adjunct in the treatment of AR.

## Introduction

Allergic rhinitis (AR) is a type 1 immediate hypersensitivity reaction resulting from an immunoglobulin E (IgE) mediated immune response on exposure to an allergen. It is estimated that 20 million to 40 million US population suffer from this condition and studies conducted in various countries reported a prevalence of rhinitis of 3% to 19% (1). Its prevalence among the school children in Kelantan, Malaysia, was 27%. The prevalence is higher in 12 years old to 14 years old age group (38.2%) than that in the 5 years old to 7 years old age group (18.2%) (2). Another study of patients newly diagnosed with AR in Malaysia showed that 10% and 21.1% had mild and moderate-severe intermittent AR, respectively, and 20% and 48.9% of patients showed mild and moderate-severe persistent AR, respectively (3). This is a long-standing disease and may affect patients’ quality of life considerably.

AR has caused significant impairments in sleep, performance at work and school, as well as difficulties in social interactions. Quality of life is significantly affected, with sleeping difficulties being the most frequently reported problem, followed by symptoms of headaches, fatigue and malaise (4). These symptoms will directly impair the patients’ concentration and productivity in the workplace, therefore, interrupting their daily activity levels (5). In children, sleep disturbance and fatigue directly impact their learning abilities and have also been implicated in behavioural disturbances (6, 7).

Treatment of AR includes allergen avoidance, antihistamine medications and intranasal steroid sprays (intranasal corticosteroid [INS]). INS is the most effective single maintenance therapy for AR that causes few side effects at the recommended doses. INS is particularly effective in the treatment of nasal congestion. Examples of INS agents include beclomethasone, flunisolide, budesonide, fluticasone propionate, mometasone furoate, fluticasone furoate and ciclesonide. Comparative studies among different glucocorticoid nasal sprays have not demonstrated significant differences in efficacy (8, 9). INS is more effective than oral antihistamines for relief of nasal blockage, nasal discharge, sneezing, nasal itchiness and postnasal drip, as demonstrated in various randomised trials and a meta-analysis (10, 11). Most studies have also favoured INS over antihistamine sprays, owing to the local anti-inflammatory effect of the corticosteroid which significantly reduces the production and activity of proinflammatory mediators such as cytokines, adhesion molecules, eosinophils and mast cells (12, 13).

Nasal irrigation is a proven adjunct in the treatment of AR. A few types of solutions can be used for nasal irrigation in AR patients, including hypotonic, isotonic or hypertonic type saline, apart from steroid nasal irrigation (14). Few studies have demonstrated that hypertonic saline is better than normal saline, possibly because of the osmotic effect of hypertonicity which induces water transport through the mucosal epithelial membrane leading to a reduction in mucosal oedema (15, 16).

Saline nasal irrigation renders mechanical cleansing of contaminants, mucus, crust and cell debris. Apart from that, it also reduces the local concentrations of pro-inflammatory mediators, humidifies the nasal mucosa and enhances mucociliary clearance (17, 18). A study by Tano and Tano (19) showed that twice-daily nasal spraying with isotonic saline significantly reduced the number of days with nasal blockage and secretion. Hypertonic nasal irrigation has been demonstrated to be beneficial in improving the quality of life in patients with sinusitis (14, 20).

The addition of steroids in nasal irrigation solution can affect the production and activity of cytokines, adhesion molecules, mast cells, and eosinophils, thus reducing the inflammation and oedema of the nasal mucosa (21). With this combined effect of mechanical cleansing and efficient steroid delivery, the use of budesonide nasal irrigation is very promising as an adjunct in the treatment of AR.

However, there is no study which looked into the effectiveness of nasal steroid irrigation in AR patients. Nonetheless, few studies had observed the effect of nasal steroid irrigation after endoscopic sinus surgery in chronic rhinosinusitis patient. Snidvongs et al. (22) in 2012 revealed that chronic rhinosinusitis (CRS) patients undergoing endoscopic sinus surgery had significantly more improvement in symptoms and endoscopy scores after receiving steroid nasal irrigation. Another study by Jang et al. (23) also concluded that the addition of budesonide nasal irrigation is helpful in the post-operative management of patients with CRS.

The purpose of this study is to describe the effects of budesonide nasal irrigation in AR patients, by comparing the Sino-Nasal Outcome Test (SNOT-22) scores, nasal endoscopy findings (turbinate hypertrophy, mucosal oedema and secretion) and serum eosinophil levels between budesonide nasal irrigation and saline nasal irrigation groups of AR patients.

## Methods

This randomised controlled study was conducted in Hospital Universiti Sains Malaysia, Kubang Kerian, Kelantan, Malaysia, from 1 November 2018 until 1 November 2019.

Subjects were recruited from Otorhinolaryngology-Head and Neck Surgery (ORL-HNS) clinic in Hospital Universiti Sains Malaysia, Kubang Kerian, Kelantan, Malaysia. The subjects with AR have the cardinal symptoms of watery rhinorrhea, bilateral nasal obstruction, nasal itchiness and excessive sneezing. Allergic rhinitis and its impact on asthma (ARIA) guideline was used to categorise the subjects according to severity and persistence of the symptoms (24). Systematic random sampling method was used. Patients who were 18 years old and above, with moderate–severe intermittent, or mild and moderate–severe persistent AR were included in the study. Patients who had a prior history of nasal surgery, patients with nasal polyps or sinonasal tumour and pregnant patients were excluded from the study.

Using the Power and Sample Size (PS) software, the calculated sample size, including 20% dropout, was 118 patients with AR, of whom half used saline nasal irrigation and the other half used budesonide nasal irrigation. The selection of patients for each group was made using a smartphone random generator application by an appointed staff nurse without the knowledge of attending doctor. All patients continued using intranasal corticosteroid (INS) and antihistamines for this research duration. The treatment duration was three months from the initiation of the treatment till the next follow-up. During the first visit, all patients underwent SNOT-22 questionnaire review, endoscopic nasal examination and blood test for eosinophil count.

For endoscopic nasal examination, the parameters assessed were the presence and severity of turbinate hypertrophy, mucosal oedema and secretions. For the blood eosinophil count, a 2 mL blood sample was taken in plain specimen bottle and sent to the pathology laboratory for processing by an assigned staff. The blood sample was analysed for full blood count and differential count, with eosinophil count. It was measured using Automated Haematology Analyser (XN-1000, S/N: 24485).

Patients were taught the proper technique of nasal irrigation using the nasal saline rinse kit (NeilMed^®^ sinus rinse). An instructional video on how to use the sinus rinse was used to assist in educating patients in proper sinus irrigation. All subject use 250 mL squeeze bottles (Sinus Rinse, NeilMed^®^ Pharmaceuticals Inc., United States) for nasal irrigation. Half of the patients used only saline solution while the other half added three puffs of budesonide inhaler (Budecort 200; Cipla Ltd., India) into the 250 mL of saline solution in the squeeze bottle. Each attenuation will deliver 200 mcg of budesonide, with a total amount of 0.6 mg per bottle. Half of the solution (125 mL) was used for each nasal cavity, irrigated once daily for 3 months. The usage of squeeze bottle was chosen as the delivery device due to studies that showed a better steroid contact with sinus mucosa while providing a small (2.5% ± 1.6%) residual fluid (25, 26).

The patients continued using the same treatment dosage for 3 months before the second assessment. After 3 months of treatment, all subjects were administered the same questionnaire, examination and investigations at the first visit. Treatment diary was used to help improve medication adherence in the patients. During follow-up visits, patients were also asked about any side effects that they experienced, such as nasal dryness, nasal itchiness or pain, epistaxis, breathing difficulty or blurring of vision. Other side effects included persistent cough, sore throat, nausea, vomiting and fever.

Data collected were entered and analysed using SPSS version 22. Descriptive statistics was used to summarise the sociodemographic characteristics of the subjects. Numerical data was presented as mean (standard deviation [SD]) or median based on their normality distribution. Categorical data was presented as frequency (%). The mean differences before and after the treatment for each group were measured using the *t*-test and the level of significance was set at 0.05. The effectiveness of budesonide nasal irrigation compared with saline nasal irrigation was displayed by measuring the difference of improvement for each group by using an independent *t*-test. The outcomes were presented in table form.

## Results

### Participants Characteristic

A total of 102 participants were recruited for this study. However, three patients defaulted the second visit and were excluded; therefore, only 99 patients remained in the study. The data obtained are expressed as mean (SD) for numerical and frequency (%) for categorical variables. The results show that the mean age of the total subjects is 39.17 and the standard deviation is 17.23. Female participants were 63.6%, while 36.4% are male. The majority are Malay (77.8%), while few are Chinese and Indian (11.1%). The intervention group (budesonide nasal irrigation) and control (saline nasal irrigation) group are evenly distributed.

**Figure 1:**
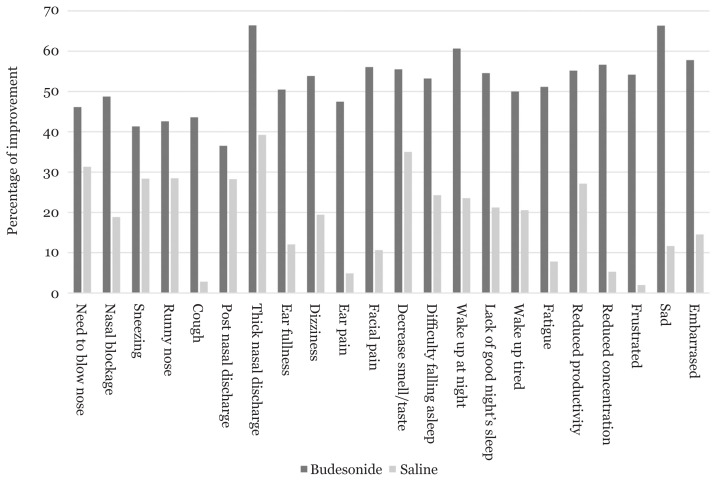
Percentage of improvement of SNOT-22 symptoms between AR patients treated with budesonide nasal irrigation and saline nasal irrigation

AR patients treated with budesonide nasal irrigation showed significant improvement in all the symptoms listed in the SNOT-22 questionnaire, while patients treated with saline nasal irrigation showed significant improvement in only a few of the symptoms (Table 1). The highest percentage of improvement for SNOT-22 symptoms is seen in thick nasal discharge in patients treated with budesonide nasal irrigation (Figure 1). There was significant improvement (*P* < 0.001) in the total symptom score of patients treated with budesonide nasal irrigation compared with those treated with saline nasal irrigation (mean improvement: 13.93; 95% CI: 8.05, 19.81) (Table 2).

There was no significant improvement of inferior turbinate hypertrophy after treatment, in patients who used either budesonide nasal irrigation or saline nasal irrigation (Table 3). However, there was a significant improvement (*P* < 0.001) of nasal mucosal oedema in patients treated with budesonide nasal irrigation compared with those treated with saline nasal irrigation (mean improvement: 0.42; 95% CI: 0.25, 0.59). In terms of nasal secretions in patients treated with budesonide nasal irrigation and saline nasal irrigation, there was a significant improvement found (*P* < 0.001) in the budesonide nasal irrigation group (mean improvement: 0.72; 95% CI: 0.51, 0.92) (Table 4).

There was no significant improvement of blood eosinophil count in both the patient groups (Table 5).

## Discussion

AR has been shown to affect patients’ quality of life considerably by reducing the quality of sleep, degrading the performance at work and school, and caused difficulties in patients’ social interactions. It is a long-standing disease with a direct impact on the healthcare costs.

In this study, we believed that combining mechanical actions of saline irrigation and local steroid effect using budesonide nasal irrigation will have added benefits in treating AR patients. Few studies were performed to look at the effects of budesonide nasal irrigation in chronic rhinosinusitis patients which yielded positive results (22, 23). However, at this moment, there is no study performed to evaluate for the effectiveness of budesonide nasal irrigation in AR patients.

This randomised controlled study looks at the effectiveness of budesonide nasal irrigation in AR patients. This was measured by comparing saline nasal irrigation with or without steroid, using three main indicators, which were symptoms (SNOT-22), clinical evaluation (via endoscopic nasal examination) and blood eosinophil levels.

SNOT-22 questionnaire reviewed the symptoms associated with AR which revealed that AR patients treated with budesonide nasal irrigation had significant improvement in all the symptoms listed in the SNOT-22 questionnaire. Whereas, patients treated with saline nasal irrigation showed significant improvement in only a few of the symptoms, namely the need to blow nose, sneezing, runny nose and thick nasal discharge.

This may be due to the effect of mechanical cleansing of saline solution. From our questionnaires, the most common symptoms are nasal blockage, sneezing, runny nose, need to blow nose and postnasal discharge. Thick nasal discharge has the highest percentage of improvement after treatment with both modalities, which was significantly better with budesonide nasal irrigation. This correlated with the clinical endoscopy findings which showed that the nasal secretions in patients treated with budesonide nasal irrigation reduced significantly.

Endoscopic nasal examination parameters assessed include inferior turbinate hypertrophy, nasal mucosal edema and nasal secretions. There was significant improvement of nasal mucosal oedema and nasal secretions in patients treated with budesonide nasal irrigation compared with those in patients using saline nasal irrigation. The reduction of nasal secretion may be due to the effect of mechanical cleansing of contaminants, mucus, crust and cell debris, which reduces the local concentrations of pro-inflammatory mediators and therefore enhances the mucociliary clearance (17, 18). The presence of steroids in the irrigation solution significantly affects the production and action of cytokines, mast cells and eosinophil. However, there was no significant improvement in inferior turbinate hypertrophy in patients using either budesonide or saline nasal irrigation.

The blood eosinophil count did not show a significant improvement after treatment in both groups. This finding may indicate that nasal irrigation, with either budesonide or saline, only exert local effects with minimal systemic effects, i.e. the blood eosinophil level. This finding also corresponds to studies which showed that the overall steroid exposure via nasal steroid irrigation is less than 5% of the total drug (24, 25). A local count of eosinophils in nasal mucosal scrapings may exhibit more valid biochemical parameters which correlate with AR (27) but due to the non-availability of these services at our centre, we used blood eosinophil levels.

## Conclusion

Budesonide nasal irrigation is effective as an adjunct in the treatment for AR. Assessment of nasal symptoms and endoscopic nasal examination revealed significant improvement after three months of treatment with this method while continuing the main treatment, which is INS. We propose that this improvement may be due to the mechanical cleansing effect which also improved the efficiency of steroid delivery to the nasal mucosa.

## Figures and Tables

**Table 1:** Comparison of SNOT-22 score between AR patients treated with budesonide nasal irrigation and saline nasal irrigation

Symptoms	Saline			Budesonide		
	
	Pre- a	Post- a	*P*-value	Pre- a	Post- a	*P*-value
1 Need to blow nose	2.52 (1.09)	1.73 (1.20)	< 0.001	2.84 (1.05)	1.53 (1.02)	< 0.001
2 Nasal blockage	2.76 (1.02)	2.24 (1.15)	0.007	3.18 (0.88)	1.63 (0.95)	< 0.001
3 Sneezing	2.82 (1.13)	2.02 (1.19)	< 0.001	3.24 (0.93)	1.90 (1.04)	< 0.001
4 Runny nose	2.74 (1.10)	1.96 (1.36)	< 0.001	3.12 (1.18)	1.79 (0.988)	< 0.001
5 Cough	1.41 (1.10)	1.37 (1.15)	0.785	1.56 (1.18)	0.88 (1.01)	< 0.001
6 Post nasal discharge	2.16 (1.36)	1.55 (1.39)	0.002	2.63 (1.33)	1.67 (1.14)	< 0.001
7 Thick nasal discharge	2.04 (1.23)	1.24 (1.09)	< 0.001	2.47 (1.23)	0.83 (0.96)	< 0.001
8 Ear fullness	1.16 (1.24)	1.02 (1.23)	0.398	2.10 (1.48)	1.04 (1.19)	< 0.001
9 Dizziness	1.44 (1.58)	1.16 (1.46)	0.113	1.82 (1.65)	0.84 (1.03)	< 0.001
10 Ear pain	0.82 (1.12)	0.78 (0.98)	0.792	1.39 (1.37)	0.73 (0.995)	< 0.001
11 Facial pain	0.94 (1.27)	0.84 (1.18)	0.294	1.48 (1.52)	0.65 (0.91)	< 0.001
12 Decrease smell/taste	1.22 (1.30)	0.78 (1.18)	0.029	1.64 (1.50)	0.73 (0.98)	< 0.001
13 Difficulty falling asleep	1.40 (1.36)	1.06 (1.42)	0.055	1.71 (1.60)	0.80 (1.15)	< 0.001
14 Wake up at night	1.36 (1.54)	1.04 (1.49)	0.058	1.55 (1.67)	0.61 (1.04)	< 0.001
15 Lack of good night’s sleep	1.32 (1.50)	1.04 (1.41)	0.142	1.65 (1.55)	0.75 (1.08)	< 0.001
16 Wake up tired	1.46 (1.46)	1.16 (1.25)	0.037	1.88 (1.45)	0.94 (1.09)	< 0.001
17 Fatigue	1.28 (1.37)	1.18 (1.33)	0.392	1.76 (1.41)	0.86 (0.94)	< 0.001
18 Reduced productivity	1.18 (1.30)	0.86 (1.23)	0.037	1.45 (1.39)	0.65 (0.89)	< 0.001
19 Reduced concentration	1.14 (1.41)	1.08 (1.37)	0.726	1.73 (1.40)	0.75 (0.93)	< 0.001
20 Frustrated	1.02 (1.44)	1.00 (1.37)	0.811	1.55 (1.44)	0.71 (1.08)	< 0.001
21 Sad	0.60 (1.13)	0.53 (1.12)	0.441	0.98 (1.22)	0.33 (0.69)	< 0.001
22 Embarrassed	0.62 (1.14)	0.53 (1.12)	0.417	0.90 (1.16)	0.38 (0.76)	< 0.001

Note:

a mean (standard deviation)

**Table 2:** Mean difference of improvement of total SNOT-22 score between AR patients treated with budesonide nasal irrigation and saline nasal irrigation

	Saline mean (SD)	Budesonide mean (SD)	Mean difference (95% CI)	*t*-statistics (df)	*P*-value
Improvement of total SNOT-22 score	−7.56 (14.78)	−21.49 (14.70)	13.93 (8.05, 19.81)	4.70 (97.00)	< 0.001

**Table 3:** Comparison of endoscopic nasal examination findings between AR patients treated with budesonide nasal irrigation and saline nasal irrigation

Endoscopic finding		Pre- a	Post- a	Mean differences a	*P*-value
IT hypertrophy	Saline	2.00 (0.00)	2.00 (0.00)	-	
	Budesonide	2.00 (0.00)	2.00 (0.00)	-	
Oedema	Saline	3.24 (0.95)	3.04 (1.02)	0.20 (−0.02, 0.42)	0.067

	Budesonide	3.47 (0.87)	2.41 (0.84)	1.06 (0.78, 1.34)	< 0.001

Secretion	Saline	2.04 (0.91)	1.76 (1.03)	0.29 (−0.03, 0.60)	0.075

	Budesonide	2.20 (0.91)	0.49 (0.87)	1.71 (1.43, 1.995)	< 0.001

Notes:

a mean (standard deviation); IT: inferior turbinate

**Table 4:** Mean difference of improvement of nasal oedema and secretion between AR patients treated with budesonide nasal irrigation and saline nasal irrigation

	Saline mean (SD)	Budesonide mean (SD)	Mean difference (95% CI)	*t*-statistics (df)	*P*-value
Oedema	−0.11 (0.38)	−0.53 (0.48)	0.42 (0.25, 0.59)	4.80 (91.26)	< 0.001
Secretion	−0.14 (0.54)	−0.86 (0.49)	0.72 (0.51, 0.92)	6.89 (97.00)	< 0.001

**Table 5:** Comparison of blood eosinophil count between AR patients treated with budesonide nasal irrigation and saline nasal irrigation

	Pre- a	Post- a	Mean differences a	*P*-value
Budesonide	0.42 (0.52)	0.33 (0.28)	0.09 (−0.02, 0.20)	0.091
Saline	0.26 (0.19)	0.25 (0.17)	0.01 (−0.02, 0.04)	0.527

Note:

a mean (standard deviation)
